# Enhanced xeno-free differentiation of hiPSC-derived astroglia applied in a blood–brain barrier model

**DOI:** 10.1186/s12987-019-0147-4

**Published:** 2019-08-29

**Authors:** Louise Delsing, Therése Kallur, Henrik Zetterberg, Ryan Hicks, Jane Synnergren

**Affiliations:** 1 Department of Neurochemistry, Institute of Neuroscience and Physiology, The Sahlgrenska Academy at the University of Gothenburg, Göteborg, Sweden; 2 Systems Biology Research Center, School of Bioscience, University of Skövde, Högskolevägen, Box 408, 541 28 Skövde, Sweden; 3 Discovery Biology, Discovery Sciences, R&D, AstraZeneca, Mölndal, Sweden; 4 BioLamina, Sundbyberg, Sweden; 5 Clinical Neurochemistry Laboratory, Sahlgrenska University Hospital, Mölndal, Sweden; 6 Department of Neurodegenerative Disease, UCL Institute of Neurology, London, UK; 7 UK Dementia Research Institute at UCL, London, UK

**Keywords:** Astroglia, hiPSC, In vitro models, Differentiation, Laminin-521, Blood–brain barrier

## Abstract

**Background:**

Human induced pluripotent stem cells (hiPSC) hold great promise for use in cell therapy applications and for improved in vitro models of human disease. So far, most hiPSC differentiation protocols to astroglia use undefined, animal-containing culture matrices. Laminins, which play an essential role in the regulation of cell behavior, offer a source of defined, animal-free culture matrix.

**Methods:**

In order to understand how laminins affect astroglia differentiation, recombinant human laminin-521 (LN521), was compared to a murine Engelbreth-Holm-Swarm sarcoma derived laminin (L2020). Astroglia expression of protein and mRNA together with glutamate uptake and protein secretion function, were evaluated. Finally, these astroglia were evaluated in a coculture model of the blood–brain barrier (BBB).

**Results:**

Astroglia of good quality were generated from hiPSC on both LN521 and L2020. However, astroglia differentiated on human LN521 showed higher expression of several astroglia specific mRNAs and proteins such as GFAP, S100B, Angiopoietin-1, and EAAT1, compared to astroglia differentiated on murine L2020. In addition, glutamate uptake and ability to induce expression of junction proteins in endothelial cells were affected by the culture matrix for differentiation.

**Conclusion:**

Our results suggest that astroglia differentiated on LN521 display an improved phenotype and are suitable for coculture in a hiPSC-derived BBB model. This provides a starting point for a more defined and robust derivation of astroglia for use in BBB coculture models.

**Electronic supplementary material:**

The online version of this article (10.1186/s12987-019-0147-4) contains supplementary material, which is available to authorized users.

## Background

Human induced pluripotent stem cells (hiPSC) can be used to generate an unlimited supply of specialized cell types from a patient’s own tissue and hold great promise to overcome issues with donor variability and low availability of human tissue. Due to the difficulties in obtaining primary human tissues from the brain, a large part of the cell biology research on neural tissues relies on stem cell-based approaches. Today, more than a decade after the discovery of hiPSC [1], well established protocols for generation of several different neural cell types are available [2–6]. However, hiPSC in vitro cultures struggle with challenges in creating an in vivo-like culture environment for the cells and low reproducibility between experiments. The use of animal-based products is still common in cell culture, even though it adds both batch-to-batch variability and chemically-undefined culture conditions. In order to improve reproducibility and quality, there is an urgent need to adapt cell cultures to defined and xeno-free conditions using only human components. This is particularly important in the field of hiPSC where large efforts are ongoing towards cell therapy applications, as cultures containing animal byproducts are subject to further regulatory requirements.

Astroglia are an abundant and diverse cell type in the human brain. In recent years, the understanding of astroglia function have transitioned from being considered as neuron helper cells to being implicated as important players in many essential brain processes and brain disorders. Glial cells have been associated with multiple pathologies involved in Parkinson’s disease (PD) [7] and Alzheimer’s disease (AD), such as tau pathology [8] and amyloid beta degradation [9]. Astroglia are also the main producers of apolipoprotein E (apoe) a major susceptibility gene associated with AD [10]. Astroglia support rapid and accurate synaptic signaling by regulating the availability of neurotransmitters, such as glutamate [11]. Glutamate regulation occurs through rapid uptake of excess glutamate, after which the glutamate can be converted to glutamine in the astroglia and recycled back to the neuron. In addition, glia cells play a key role in the formation and maintenance of the blood–brain barrier (BBB) through physical interaction with the brain microvascular endothelial cells and secretion of modulating factors [12–16]. The BBB is mainly composed of brain microvascular endothelial cells (BMEC), astrocytic endfeet, pericytes and the basement membrane (BM). The BMEC have specific properties that allow them to restrict permeability between the blood and the brain. The tight cellular interactions between the BMEC in the BBB act as a physical barrier for pathogens, cells, proteins and water-soluble agents. Specific transport proteins control the supply of nutrients and the transfer of other small molecules to the brain. The BM contains specific, highly conserved proteins, and consists mostly of laminin, type IV collagen, agrin, perlecan, fibronectin and nidogen [17].

Laminins are the most abundant component of the BM. In addition to their structural functions, laminins play essential roles in the organization of the BM and in the regulation of cell behavior [18]. Laminins are multidomain, heterotrimeric glycoproteins, composed of three different subunits; an α-chain, β-chain and γ-chain, combined and expressed in at least 16 different isoforms in the human body [19]. The physical, topological, and biochemical expression of the different laminin isoforms in the BM is heterogeneous, and laminin expression changes during development. Consequently, without the right combination of laminin isoforms, cells and tissues become dysfunctional. BMECs generate laminins-411 and -511 whereas astrocytes produce laminins-111, -211 and -521. All these laminin isoforms are also expressed by the primary brain capillary pericytes [20–22].

Using specific laminin isoforms to mimic physiologically relevant extra cellular matrices has previously been shown to have beneficial effects on both cell culture and differentiation of several cell types including hepatocytes, retinal pigment epithelium, keratinocytes, dopamine and pancreatic cells [23–26]. Specifically, alpha 5 laminins (laminins-511 and -521) enhance functional development of hiPSC-derived neurons and support network formation [27], are essential for astroglia migration and vessel formation in the retina [28], and play a key role in the maintenance of BBB integrity [29]. To date, most differentiation protocols for hiPSC-astroglia use undefined reagents, containing animal-based products. Usually the culture surfaces are coated with a BM mixture produced by murine Engelbreth-Holm-Swarm sarcoma [30–33], which contain primarily laminin-111 as well as other BM proteins and unknown substances secreted by the sarcoma cells. In vitro hiPSC-derived BBB models need authentic astroglia to produce a brain-like microenvironment for the endothelial cells in order to function properly and serve as a true predictive model [34–39]. Adapting astroglia differentiation protocols to defined and xeno-free conditions is desirable, and a detailed understanding of how specific laminin isoforms affect both the differentiation and maturation of astroglia is essential for in vitro applications in both astroglia models and in BBB models.

To adapt astroglia differentiation towards defined conditions and identify how differentiation on astrocyte-specific laminin-521 affects hiPSC-derived astroglia and the use of these astroglia in BBB models, we compared the differentiation of three different hiPSC lines to astroglia. Long-term neuroepithelial stem cells (NES) were generated from three different hiPSC lines and then comparatively differentiated from NES to astroglia on human laminin-521 (LN521) and murine Engelbreth-Holm-Swarm sarcoma laminin (L2020). Astroglia protein expression and function were evaluated. Finally, we investigated the functionality of these cells in a coculture setting where the astroglia were cocultured with endothelial cells in an in vitro model of the BBB.

## Methods

### Cells

hiPSC lines from three different healthy donors, C1 [40], C9 [41] and AF [42] were used in all astroglia differentiation experiments. Data presented are mean values and standard deviations across these three cell lines. Brain endothelial cells were derived from the previously-described hiPSC line r-iPSC1J [43], these brain endothelial cells are referred to as r-iBECS.

### Differentiation

hiPSC lines were differentiated to NES and then to astroglia using our previously published protocol [3]. NES cells were differentiated to astroglia in plates coated with poly-l-ornithine and murine L2020 (Sigma Aldrich, St. Louis, MO, USA) or LN521 (Biolaminin 521, BioLamina, Sundbyberg, Sweden) at 2 µg/ml.

### Proliferation

Cell proliferation was measured using an IncuCyte S3 Live-Cell Analysis System (Essen BioScience, Ann Arbor, MI, USA). The IncuCyte S3 monitors proliferation in a label free manner by analyzing the occupied area (confluence %) of cell images over time. As cells proliferate, the confluence increases. Proliferation is presented as confluence increase, in percent of well area per hour.

### Immunocytochemistry

Immunocytochemistry and staining were carried out after fixing the cells in 4% formaldehyde for 10–20 min at room temperature. The fixed cells were washed using phosphate-buffered saline (PBS) solution and incubated in a blocking and permeabilization buffer; 10% FBS, 0.1% Triton X, PBS (all from Invitrogen, Carlsbad, CA, USA), for 1 h at room temperature. Primary antibodies were diluted in antibody buffer; 4% FBS, 0.01% Triton X, PBS (all from Invitrogen), and incubated with the cells at 4 °C overnight. Appropriate secondary antibodies were used in mono labeling. For primary and secondary antibodies used in the study see Additional file 1: S1, Table of Antibodies. DAPI (1:2000; Invitrogen) was used for nuclei staining. Images were captured using an ImageXpress wide field microscope, and downstream image analysis to obtain percent positive cells utilized MetaXpress software (both from Molecular Devices, Sunnyvale, CA, USA).

### qPCR

A minimum of 200,000 cells were collected and RNA was purified using the RNeasy Mini Kit (Qiagen, Hilden, Germany) with DNase treatment according to the manufacturer’s instructions. RNA was reverse transcribed using the High-Capacity cDNA Reverse Transcription kit (Applied Biosystems, Foster City, CA, USA). cDNA amounts were detected using TaqMan gene expression assays (Applied Biosystems) on a 7900HT Sequence Detection System (Applied Biosystems). For specification of assays used see Additional file 1: S2, Table of qPCR assays. Expression data were analyzed and related to the level of GAPDH using the dCt method [44].

### Glutamate uptake

A Glutamine/Glutamate Determination Kit (Sigma Aldrich) was used to measure the decrease of glutamate in the cell culture media over time. Assays were initiated 72 h after plating in order to let the cells recover. Cells were seeded at 70,000 cells/cm^2^. Before the assay cells were washed with HBSS (Invitrogen) buffer and incubated with HBSS (Invitrogen) for 30 min. To evaluate the contribution of EAAT1, 100 µM l-glutamic acid (Invitrogen) was prepared with the EAAT1 inhibitor UCPH 101 (Abcam, Cambridge, UK) at 1.34 µM, the solution was prepared in HBSS (Invitrogen) with or without inhibitor and incubated with the cells. After 60 min the remaining glutamate concentration in the media was measured following enzymatic reaction at 340 nm using a Multi-label reader (Perkin Elmer, Waltham, MA, USA). After subtraction of background (blank sample containing 0 nM glutamate), the decrease of glutamate in the media was determined using the glutamate standard prepared according to manufacturer’s instructions. Directly after sample isolation, analysis of double stranded DNA content in each well was performed with Quant-iT™ PicoGreen™ dsDNA Reagent (Life Technologies, Carlsbad, CA, USA), according to the manufacturer’s instructions. The double stranded DNA content was used to normalize glutamate uptake data.

### Protein secretion

Cells were seeded in T25 flasks (Corning, Corning, New York, USA) at 50,000 cells/cm^2^. After 48 h, 5 ml media samples were collected from the flasks and centrifuged at 1000*g* for 5 min. The supernatant was collected and concentrations of GDNF, APOE, S100B, Angiopoietin-1, IL6 and IL8 were analyzed using commercially available immunoassays. S100B concentration was measured using an Elecsys assay on a Cobas instrument (Roche Diagnostics, Basel, Switzerland). IL-8 concentrations were measured using a multiplexed immunoassay with electrochemiluminescence detection (Meso Scale Discovery, Rockville, MD, USA). ApoE concentration was measured using an enzyme-linked immunosorbent assay (Mabtech, Nacka Strand, Sweden). Angiopoietin-1, GDNF, and IL6 concentration were measured using an enzyme-linked immunosorbent assays (Merck, Kenilworth, NJ, USA).

### BBB model

Brain endothelial cells were derived from hiPSC using a previously published protocol [36] using human serum instead of bovine. Astroglia were seeded at 40,000 cells/cm^2^ 2 days before the start of coculture. Endothelial cells were seeded at 1,000,000 cells/cm^2^ on collagen (400 µg/ml)/fibronectin (100 µg/ml) coated 24 well 0.4 µm pore polyester membrane transwell inserts (Corning) and allowed to attach for at least 6 h. Endothelial cell layer integrity was confirmed by visual inspection and TEER measurement, any transwells with TEER lower than 800 Ω/cm^2^ were not used in subsequent experiments. Coculture was initiated by changing the media in the astroglia culture to 1 ml endothelial media and inserting the transwell membrane with endothelial cells. BBB property analyses were performed 3 days after initiation of the coculture.

### Transendothelial electrical resistance measurements

Transendothelial electrical resistance (TEER) measurements were carried out using an EVOM2 Epithelial Voltohmmeter (World Precision Instruments, Sarasota, FL, USA). The resistance value was calculated using the equation below. Empty filters coated with collagen/fibronectin were used as blanks. All TEER measurements were performed in triplicates.

$${\text{TEER }}\left( {\Omega \times {\text{cm}}^{ 2} } \right) = \left( {{\text{TEER}}\left( {\text{Endothelial cells}} \right)-{\text{TEER}}\left( {\text{blank}} \right)} \right) \times {\text{Area of culture}}$$

### Fluorescein permeability

Cells were washed with HBSS (Life Technologies) before the addition of sodium fluorescein (Sigma Aldrich) at 1 µM in HBSS to the apical chamber and HBSS to the basolateral chamber. Cells were incubated on a rotating platform for 60 min at 37 °C. Fluorescein concentration in the basolateral compartment was calculated after measuring fluorescence on a plate reader (485 nm excitation and 535 nm emission).

### Statistical analysis

Student’s t-test and two-way ANOVA statistical analysis were used.

## Results

### Astroglia specific protein and mRNA expression

**Fig. 1:**
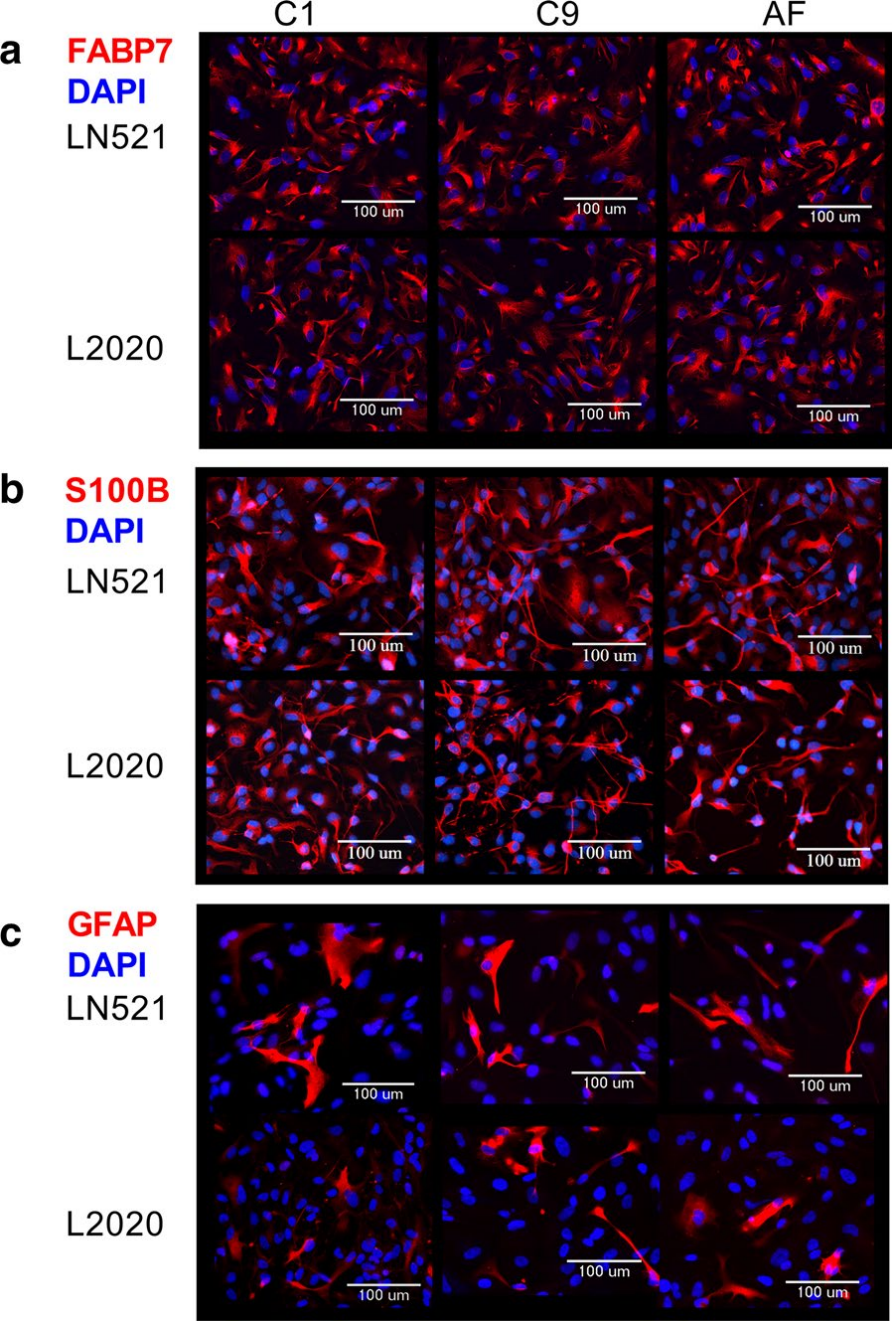
Characterization of astroglia associated protein expression in hiPSC astroglia differentiated on recombinant human laminin-521 (LN521) and murine laminin (L2020). **a**–**c** Representative immunocytochemistry images of three iPSC lines, C1, C9 and AF differentiated to astroglia on LN521 or L2020. Astroglia differentiated on L2020 and LN521 showed expression of fatty acid binding protein (FABP7) (**a**), S100B (**b**) and partial expression of glial fibrillary acid protein (GFAP) (**c**)

**Fig. 2:**
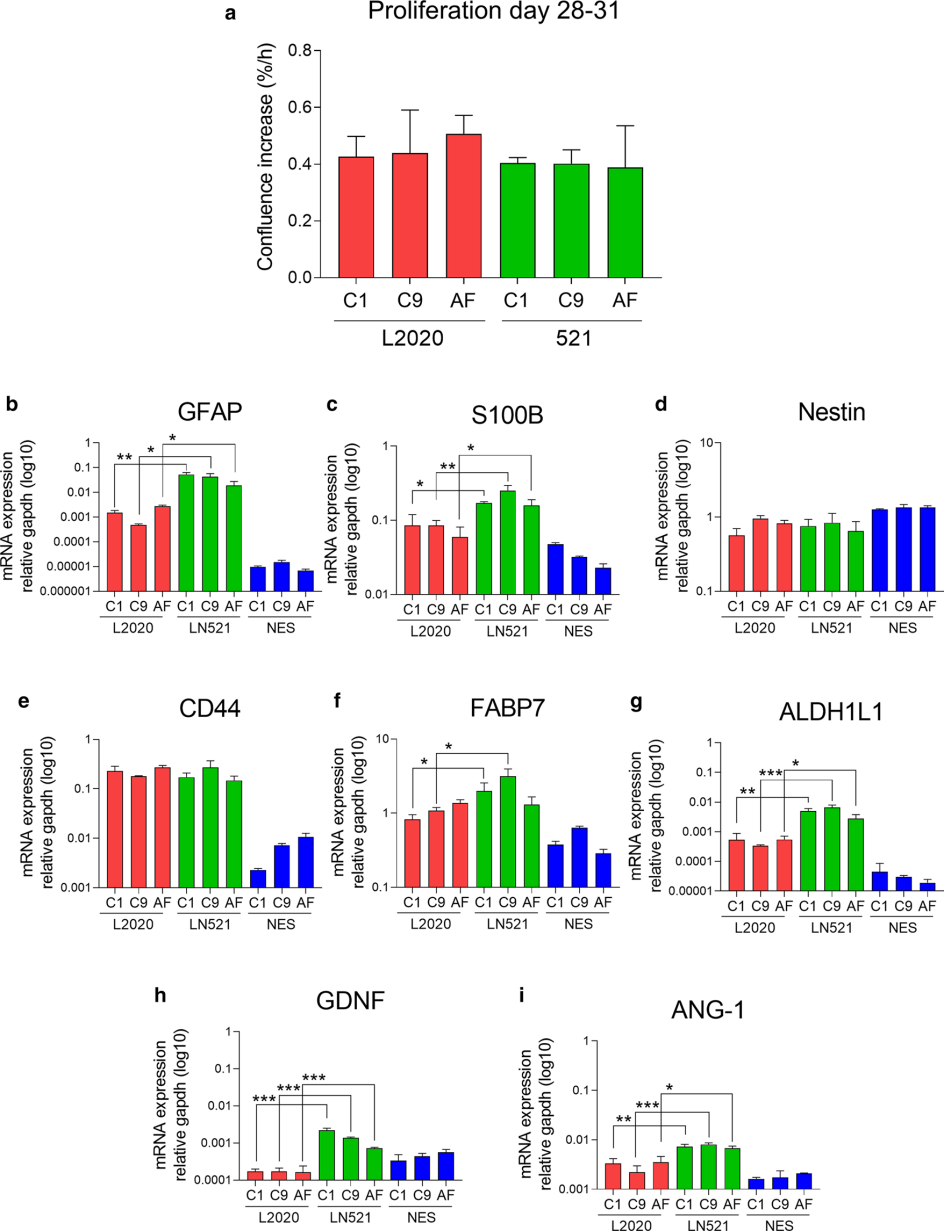
Characterization of proliferation and mRNA expression of astroglial marker mRNA in hiPSC astroglia differentiated on recombinant human laminin-521 (LN521) and murine laminin (L2020). **a** Proliferation rate analysis showed no differences between astroglia differentiated on LN521 and L2020. **b**–**i** Expression analysis of astroglia related mRNA, GFAP (**b**), S100B (**c**), Nestin (**d**), CD44 (**e**), FABP7 (**f**), aldehyde dehydrogenase (ALDH1L1) (**g**), glial derived neurotrophic factor (GDNF) (**h**) and Angiopoietin-1 (Ang-1) (**i**). mRNA expression of GFAP, S100B, ALDH1L1, GDNF and Ang-1 were higher in astroglia differentiated on LN521 compared to astroglia differentiated on L2020 for all lines. The C1 and C9 lines differentiated on LN521 had higher expression of FABP7 compared to differentiation on L2020. NES included as reference. Significance indicated by *p < 0.05, **p < 0.01 ***p < 0.001

Astroglia differentiated on human LN521 or murine L2020 were characterized based on morphology and astroglia marker expression after 28 days of differentiation. Immunostaining analysis showed that astroglia differentiated on LN521 and L2020 expressed glia markers, such as fatty acid binding protein 7 (FABP7) (Fig. 1a), calcium binding protein S100B (Fig. 1b) and partially expressed glial fibrillary acid protein (GFAP) (Fig. 1c). Analysis of proliferation rate showed no difference between astroglia differentiated on LN521 compared to L2020 (Fig. 2a). Analysis of the expression of astroglia marker genes (Fig. 2b–i) revealed higher expression of several mRNAs in astroglia differentiated on LN521 when compared to L2020. LN521 differentiated astroglia showed higher expression of glia fibrillary acid protein (GFAP), S100B, aldehyde dehydrogenase (ALDH1L1), glial derived neurotrophic factor (GDNF) and angiopoietin-1 (Ang-1) for all three lines. Additionally, the C1 and C9 lines showed higher expression of fatty acid binding protein 7 (FABP7) in astroglia differentiated on LN521 compared to L2020. Taken together, astroglia differentiated on LN521 displayed differences in mRNA expression of several astroglia marker mRNAs compared to astroglia differentiated on L2020.

### Glutamate metabolism

**Fig. 3:**
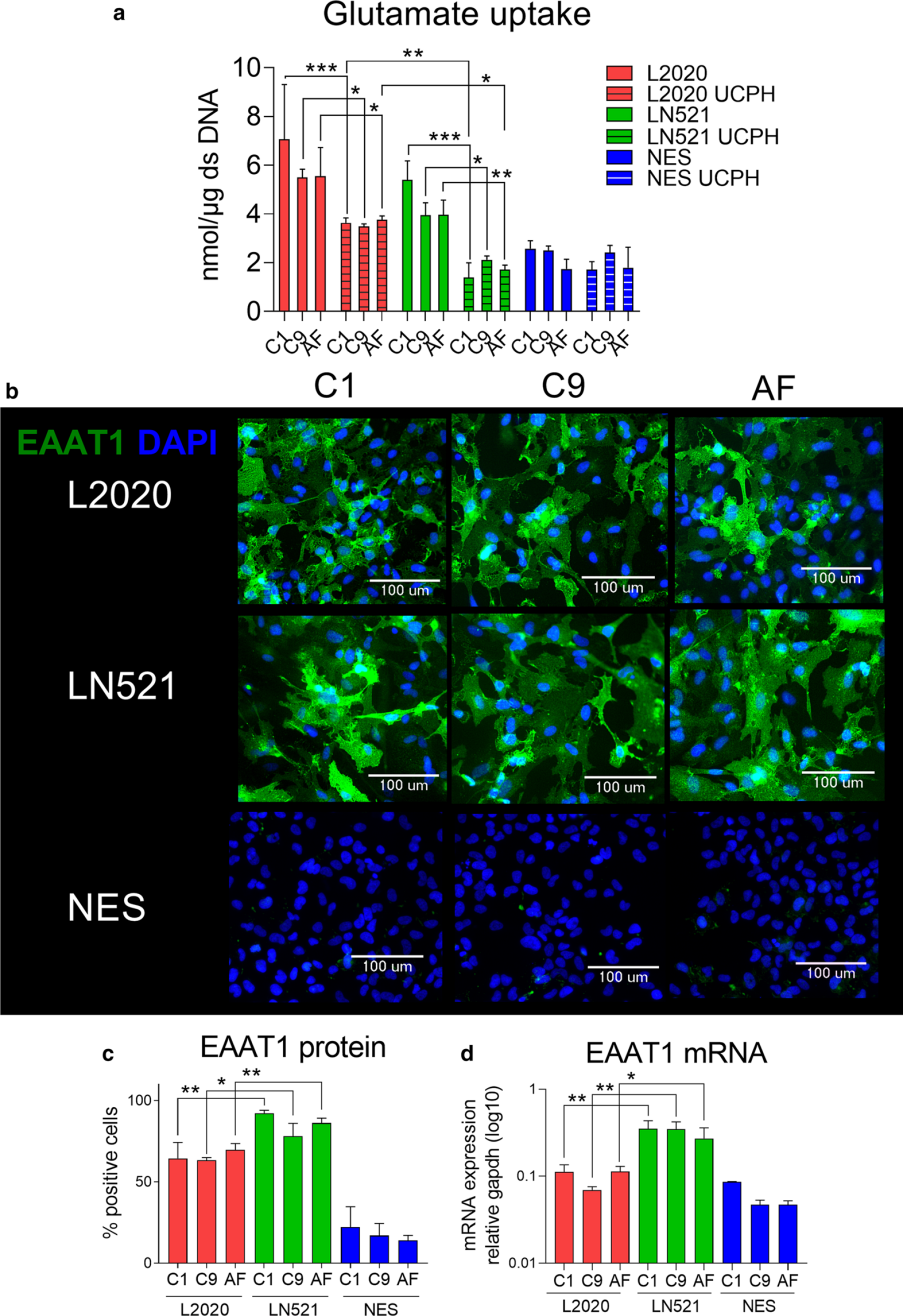
Glutamate uptake compared between hiPSC astroglia differentiated on human, recombinant laminin-521 (LN521) and murine laminin (L2020). **a** Astroglia uptake of glutamate in the presence and absence of the EAAT1 inhibitor UCPH101 (UCPH). Astroglia differentiated on both LN521 and L2020 have glutamate uptake, which is inhibited by UCPH 101. C1 and AF astroglia differentiated on LN521 show a larger reduction in glutamate uptake when treated with the inhibitor UCPH. **b** Representative immunocytochemistry images show that both astroglia differentiated on LN521 and L2020 express EAAT1. **c** Image analysis of EAAT1 expression showed that the percentage of cells expressing EAAT1 was higher in astroglia differentiated on LN521 compared to L2020. **d** mRNA analysis of EAAT1 showed that astroglia differentiated on LN521 had higher expression of EAAT1 mRNA, compared to astroglia differentiated on L2020. Neuro epithelial stem cells (NES) included as reference. Significance indicated by *p < 0.05, **p < 0.01, ***p < 0.001

Maintaining the signaling capacity of the synapse by removing excess glutamate from the synaptic cleft is one of the main functions of astroglia in the brain. Astroglia perform this task by efficient glutamate uptake into the cell. In astroglia, glutamate uptake occurs mainly through EAAT1 and EAAT2. mRNA levels of EAAT1 were higher in astroglia differentiated on LN521 compared to L2020 while no difference in EAAT2 mRNA expression was observed. To evaluate functional glutamate uptake by EAAT1 in hiPSC-derived astroglia, glutamate uptake was measured in the presence and absence of the EAAT1 inhibitor UCPH101 (Fig. 3a). Astroglia differentiated on both LN521 and L2020 had functional glutamate uptake that was inhibited by UCPH101, however, the reduction in glutamate uptake upon inhibition of EAAT1 was greater in astroglia from the C1 and AF lines differentiated on LN521 than on L2020. Image-based quantification of EAAT1 staining (representative images Fig. 3b) showed that the fraction of cells expressing the EAAT1 protein (Fig. 3c) was significantly higher in astroglia differentiated on LN521 (means ± standard deviations: C1 92.2 ± 1.4, C9 78.1 ± 6.0, AF 86.1 ± 2.4) than in astroglia differentiated on L2020 (means ± standard deviations: C1 64.4 ± 8.0, C9 63.3 ± 1.3, AF 69.6 ± 3.2). Similarly, EAAT1 mRNA was more highly expressed in astroglia differentiated on LN521 than on L2020, for all lines (Fig. 3d).

**Fig. 4:**
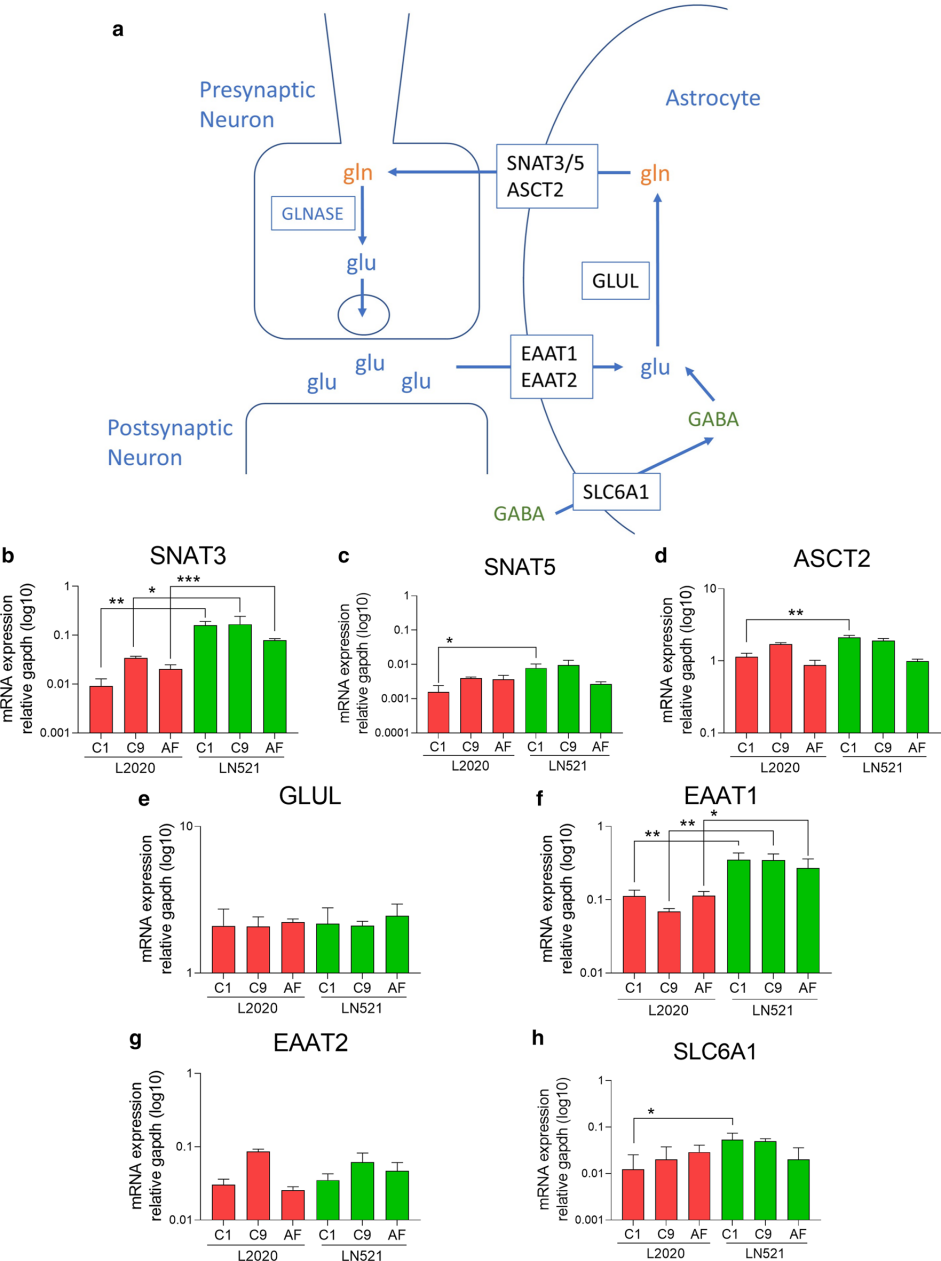
Glutamate metabolism related mRNA expression in hiPSC astroglia differentiated on human, recombinant laminin-521 (LN521) or murine laminin (L2020). **a** A schematic overview of glutamate uptake, conversion to glutamine and glutamine export in astroglia. **b**–**h** mRNA expression of glutamine exporters, SNAT3 (**b**), SNAT5 (**c**), ASCT2 (**d**), glutamine synthase (GLUL) (**e**), glutamate importers EAAT1 (**f**) and EAAT2 (**g**), GABA importer SLC6A1 (**h**). mRNA expression of SNAT3 and EAAT1 was higher in astroglia differentiated on LN521 compared to L2020, for all three hiPSC lines. The C1 line differentiated on LN521 additionally showed higher expression of SNAT5, ASCT2 and SLC6A1 compared to C1 differentiated on L2020. Significance indicated by *p < 0.05, **p < 0.01, ***p < 0.001

To further understand these differences in glutamate uptake seen in astroglia differentiated on LN521 and L2020, mRNA levels of genes encoding proteins involved in the glutamate metabolism (Fig. 4a) in astroglia were evaluated. mRNA levels of glutamine exporter SNAT3 and EAAT1 (Fig. 4b, f) were significantly higher in astroglia differentiated on LN521 than astroglia differentiated on L2020, in all lines. In addition, the C1 line astroglia showed higher expression of SNAT5, ASCT2, and SLC6A1 (Fig. 4c, d, h) when differentiated on LN521 compared to differentiation on L2020. No other differences were observed in mRNA levels of glutamine exporters SNAT5 and ASCT2, glutamine synthase GLUL, glutamate importer EAAT2 and GABA transporter SLC6A1 (Fig. 4c–e, g, h). These results imply that differentiation on LN521 affects the glutamate metabolism as astroglia differentiated on LN521 showed higher expression of both glutamate importer EAAT1 and glutamine exporter SNAT3 across all the three lines.

### Secretion of astroglia related proteins

**Fig. 5:**
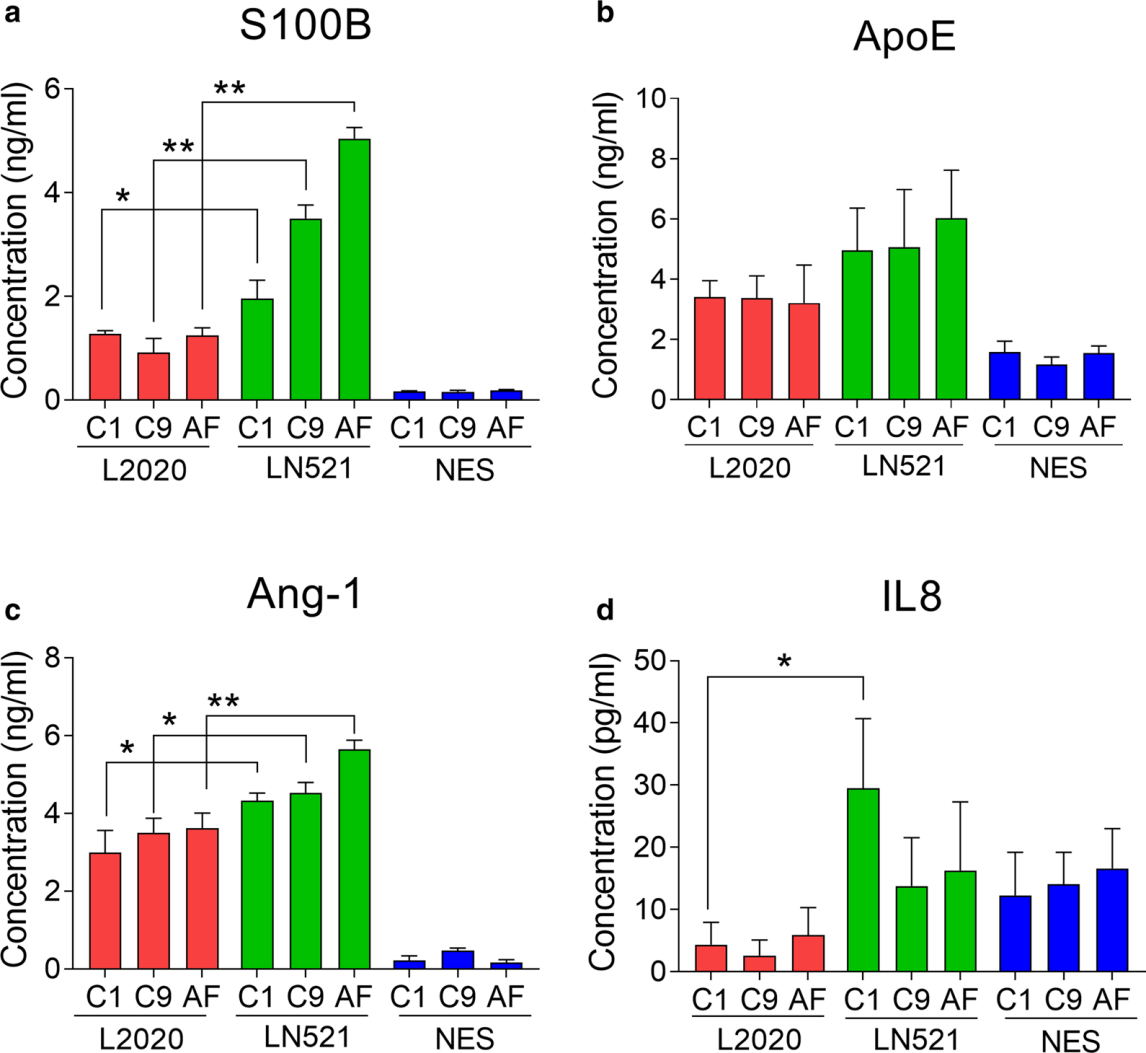
Protein secretion by hiPSC-derived astroglia differentiated on human, recombinant laminin-521 (LN521) or murine laminin (L2020). **a**–**d** Concentrations of S100B (**a**), ApoE (**b**), angiopoietin-1 (Ang-1) (**c**) and IL8 (**d**) were measured in media collected from astroglia differentiated on LN521 or L2020. Secretion of S100B and Ang-1 to the media, were higher in astroglia differentiated on LN521 than astroglia differentiated on L2020, for all lines. The C1 line differentiated on LN521 had higher secretion of IL8 compared to C1 differentiated on L2020. Significance indicated by *p < 0.05, **p < 0.01, ***p < 0.001

Astroglia are the most abundant cell type of the human brain and proteins secreted by astroglia have many important functions in the nervous system, particularly in regulating the BBB. Secretion of IL8 and IL6 were investigated to understand if the inflammatory state of the astroglia is different between differentiation on LN521 or L2020. L2020 could contain unknown substances secreted by the murine sarcoma cells that effect interleukin secretion. To understand if the culture matrix had effects on selected secreted proteins, media concentrations of S100B, APOE, Ang-1, IL8, IL6, and GDNF were measured at day 28 of differentiation (Fig. 5a–d). Media concentrations of GDNF and IL6 were very low, below 2.7 pg/ml and 4 pg/ml respectively in all samples (data not shown). Astroglia differentiated on LN521 secreted more S100B (Fig. 5a) and Ang-1 (Fig. 5c) into the media compared to astroglia differentiated on L2020 for all lines. IL8 secretion was higher in C1 astroglia differentiated on LN521 than L2020, however, this difference was not seen in the other lines (Fig. 5d). No significant differences in media concentrations of APOE were observed between astroglia differentiated on LN521 and L2020 (Fig. 5b).

### BBB induction of hiPSC-derived endothelial cells

**Fig. 6:**
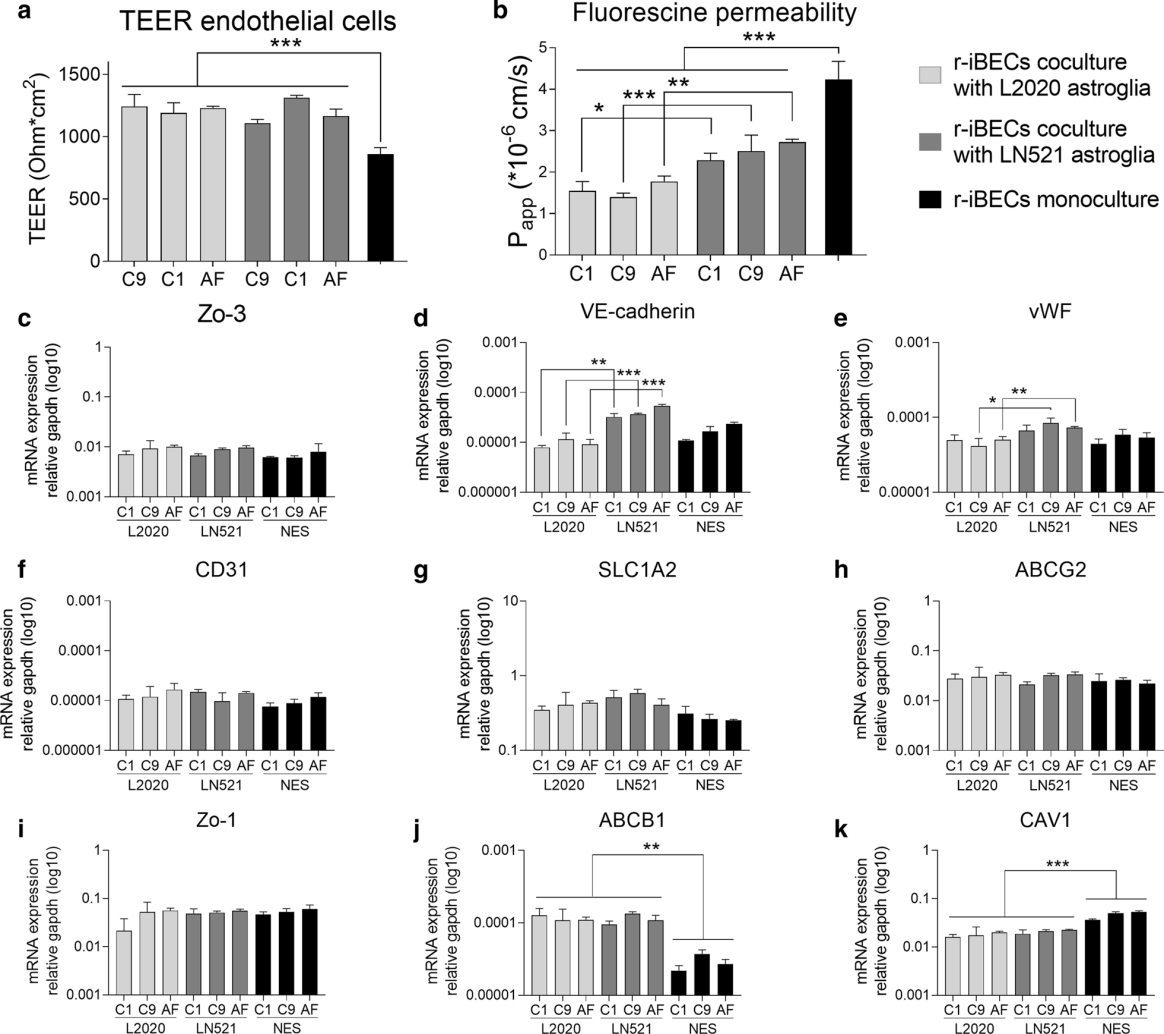
In vitro modelling of the blood–brain barrier (BBB) using hiPSC-derived brain endothelial cells (r-iBECs) cocultured with hiPSC-derived astroglia differentiated on human, recombinant laminin-521 (LN521) or murine laminin (L2020). **a** Paracellular permeability as measured by TEER showed that endothelial cells have higher TEER in coculture with both astroglia differentiated on LN521 and L2020 as compared to monoculture. **b** Permeability of fluorescein was decreased in r-iBECs cocultured with both astroglia differentiated on LN521 and L2020 compared to monoculture. In addition, fluorescein permeability was lower in r-iBECs in coculture with astroglia differentiated on L2020 compared to LN521. **c**–**k** Characterization of r-iBECs by mRNA expression levels of BBB associated genes in monoculture or coculture with astroglia. mRNA levels of tight junction protein 3 (TJP3) (**c**), adherence junction protein VE-cadherin (**d**), endothelial marker vWF (**e**), adherence junction protein CD31 (**f**), glucose transporter SLC1A2 (**g**), efflux transporter ABCG2 (**h**), tight junction protein 1 (TJP1) (**i**) and efflux transporters ABCB1 (**k**) were analyzed. Expression levels of VE-cadherin mRNA were observed to be higher in endothelial cells cocultured with astroglia differentiated on LN521 compared to L2020 for all lines. mRNA levels of vWF were higher in r-iBECs cocultured with astroglia derived from the C9 and AF lines differentiated on LN521 compared to differentiation on L2020. NES included as reference. Significance in Significance indicated by *p < 0.05, **p < 0.01, ***p < 0.001

In vitro, the barrier function in brain endothelial cells is promoted by astroglia cocultures. Coculture BBB models were set up with hiPSC-derived astroglia and hiPSC-derived brain endothelial cells (r-iBECs). Barrier properties of the endothelial cells in coculture with either astroglia differentiated on LN521 or on L2020 were compared (Fig. 6). Coculture with both LN521 astroglia and L2020 astroglia increased the paracellular tightness of r-iBECs as measured by TEER, compared to monocultured r-iBECs, for all lines (Fig. 6a, Table 1). No differences were found in induction of TEER between LN521 astroglia and L2020 astroglia. Passive permeability measured by fluorescein permeability was decreased in r-iBECs cocultured with both LN521 astroglia and L2020 astroglia compared to monoculture r-iBECs. Fluorescein permeability was lower in r-iBECs cocultured with L2020 astroglia compared to coculture with LN521 astroglia (Fig. 6b, Table 2). mRNA analysis of r-iBECs cocultured with either LN521 astroglia or L2020 astroglia (Fig. 6c–k) showed higher expression of VE-cadherin in r-iBECs cocultured with LN521 astroglia lines compared to r-iBECs cocultured with L2020 astroglia lines (Fig. 6d). r-iBECs cocultured with C9 and AF LN521 astroglia showed higher expression of von Willebrand factor (vWF) compared to r-iBECs cocultured with L2020 astroglia lines (Fig. 6e). No other differences between coculture with astroglia differentiated on LN521 and L2020 were observed.

**Table 1:** TEER r-iBECs in coculture and monoculture (Ω cm^2^)

	Mean TEER	Standard dev.
r-iBECs in coculture with L2020 astroglia		
C1	1191.6	66.4
C9	1243.5	77.9
AF	1229.9	12.4
r-iBECs in coculture with LN521 astroglia		
C1	1312.6	16.7
C9	1109.7	24.5
AF	1166.4	45.1
r-iBECs in monoculture	858.8	44.1

**Table 2:** Fluorescein permeability of r-iBECs in coculture and monoculture (*10^−6^ cm/s)

	Mean P_app_	Standard dev.
r-iBECs in coculture with L2020 astroglia		
C1	1.5	0.2
C9	1.4	0.1
AF	1.8	0.1
r-iBECs in coculture with LN521 astroglia		
C1	2.3	0.1
C9	2.5	0.3
AF	2.7	0.1
r-iBECs in monoculture	4.2	0.4

Interestingly, differences were observed between coculturing with differentiated astroglia (LN521 and L2020) compared to NES, for all lines. The increase in expression of ABCB1 and the decreased expression of caveolin 1 (Cav1) in r-iBECs appeared to depend on coculture with differentiated astroglia and was not observed in coculture with NES (Fig. 6j, k). In conclusion, astroglia differentiated on LN521 and L2020 exhibited slightly different effects on r-iBECs in coculture.

## Discussion

In order to initiate adaptation of astroglia differentiation to xeno-free conditions we compared differentiation on recombinant human LN521 or murine L2020 and evaluated these cells in a BBB model. In general, astroglia differentiated on LN521 and L2020 showed similar characteristics and the experiments show that it is possible to derive high-quality astroglia on both LN521 and L2020. This result provides an important starting point for the adaptation of astroglia differentiation protocols to good manufacturing practice (GMP) compliant standards. However, highly interesting differences in astroglia properties were observed between using LN521 and L2020 as differentiation matrices. Astroglia differentiated on LN521 showed higher mRNA expression of known astroglia markers such as GFAP, GDNF, and S100B, across all three iPSC lines included in this study.

The inflammatory state of astroglia could affect coculture BBB models as both IL8 and IL6, known to be secreted by astrocytes [45], have been shown to affect the permeability of endothelial cells in vitro at ng/ml concentrations [46, 47]. The analyses of secreted protein levels show that IL6 secretion was very low and that variability in IL8 secretion was high. The C1 line showed higher IL8 secretion when differentiated on LN521 compared to L2020 while no differences were identified in IL8 secretion in the other lines, and hence no uniform changes were observed in IL8 and IL6 secretion across the three cell lines. Low production of both IL6 and IL8 are observed in normal astroglia in a resting state [45] and the concentrations observed in this study are substantially lower than what has been shown to affect the BBB permeability. Hence, it is unlikely that the interleukins secreted by astroglia in these experiments are affecting r-iBECs in coculture, however, it remains elusive if there is a difference in the inflammatory state between astroglia differentiated on LN521 and L2020.

Different subtypes of astroglia show different astroglia marker expression profiles. Protoplasmic astroglia express S100B but not GFAP, which is mainly expressed in fibrous and reactive astroglia [48]. The protein expression profiles displayed by astroglia derived in this study suggest that they are of mixed subtypes with mainly protoplasmic phenotype. Astroglia differentiated on LN521 have higher secretion of S100B and Ang-1 compared to astroglia differentiated on L2020. These results are in agreement with increases in mRNA levels of Ang-1 and S100B. S100B is widely used as a marker for astroglial identity and is expressed at low levels in neural stem cells. The expression of S100B is increasing throughout the differentiation to astroglia, as astroglia become more mature [3, 49]. As an extracellular signal, S100B protects neurons against apoptosis, stimulates neurite outgrowth and astroglia proliferation, and negatively regulates astrocytic and microglial responses to neurotoxic agents [50]. S100B has a dual role as a neurotrophic factor and as a neurotoxic factor and its effects have been shown to depend on the protein concentration, with doses of S100B up to a few hundred nM being neurotrophic and higher doses being neurotoxic [51]. Physiological extracellular concentrations of S100B have been estimated to be a few nM [50]. S100B secreted from astroglia in this study (recalculated to nM) were approximately 0.1 nM in L2020 cultures and 0.5 nM in LN521 cultures. Astroglia cultured on LN521 have higher S100B mRNA expression and higher secretion of S100B suggesting that there is an increase in both S100B mRNA and S100B protein levels. Even though these hiPSC-derived astroglia are still immature, their secretion of S100B is approaching physiological levels. Consequently, astroglia differentiated on LN521 may display an increased maturity compared to astroglia differentiated on L2020. Previous studies in mice have shown that alpha 5 laminins are important culture substrates for astroglia [28]. Furthermore, alpha 5 laminins are produced in high levels by endothelial cells in the brain [20], and given the close proximity and interaction of astroglia and brain endothelial cells [12, 52] astroglia are in contact with alpha 5 laminins in vivo. Hence, LN521 may create an environment for the astroglia that is more similar to the in vivo situation, which may contribute to the observed differences.

Glutamate uptake related proteins were affected by the culture matrix used to differentiate astroglia. Astroglia cultures differentiated on LN521 were found to have higher expression of EAAT1 at both mRNA and protein levels, in all three investigated lines. In addition, these astroglia expressed higher mRNA levels of glutamine exporter SNAT3. The increase in the population expressing EAAT1 protein and mRNA levels were not reflected in the total glutamate uptake of the cells but for the C1 and AF lines, the reduction in uptake after inhibition of EAAT1 was greater in astroglia differentiated on LN521. It is likely that other transport routes than EAAT1 are contributing to the glutamate uptake in the astroglia population. Both due to the relative immaturity of iPSC-derived astroglia and the mixed population with approximately 15% of cells not expressing EAAT1 on LN521. Hence it is possible that the total uptake is not different but the fraction of the uptake that goes through EAAT1 has increased when the expression of EAAT1 is increased. The major part of astroglia maturation occurs after birth [53]. In the rodent postnatal brain, glutamate uptake activity mainly depends on EAAT1, and EAAT1 activity increases during the 1st weeks after birth [54, 55]. Hence, the increase in EAAT1 expression seen in astroglia differentiated on LN521 could represent a more mature postnatal astroglia phenotype. After the first postnatal weeks, EAAT2 activity is increased in a neuron dependent manner [56], and in the adult brain, the EAAT2 activity is responsible for the majority of the glutamate uptake [57]. Consequently, future efforts towards deriving astroglia from hiPSC showing a more adult glutamate uptake phenotype could benefit from coculturing with neurons.

When interpreting the results, it is important to keep in mind that the data is not a complete evaluation of differential gene expression dependent either on LN521 or L2020. Rather it is an analysis of several astroglia-associated genes and proteins, which are strongly linked to astroglia identity and functionality, chosen to provide an assessment of how the differentiated cells compared to each other. Furthermore, it cannot be excluded that the observed differences are, at least partly, influenced by the higher proportion of intact full-length laminin molecules in the recombinant LN521 laminin or the presence of unknown substances produced by sarcoma cells in the murine L2020.

To characterize the astroglia in an in vitro cell model application, we compared their ability to induce barrier properties in r-iBECs. Astroglia differentiated on both LN521 and L2020 create coculture BBB models with high TEER and low fluorescein permeability in the range of 1.5–3 × 10^−6^ cm/s, comparable to previously published hiPSC-derived BBB coculture models [34, 38, 39, 58]. LN521 and L2020 astroglia promoted expression of BBB-specific proteins and transporters, and LN521 astroglia cocultures showed to improve the expression of VE-cadherin in r-iBECs. Notably, expression of VE-cadherin was one of the improvement points previously identified for this model [39] and our data suggest that this can be achieved by using astroglia differentiated on LN521. Previous reports show that growth factors secreted by astroglia, such as GDNF and Ang-1, contribute to increased barrier tightness and tight junction stability. Ang-1 increases survival in several endothelial cell types through the Pi3-Akt pathway [59, 60]. In addition, Ang-1 has been shown to affect permeability in endothelial cells through the strengthening of junctions [61, 62] and to attenuate harmful effects of thrombin by stabilizing Zo-1 and occludin [63]. The addition of GDNF to in vitro brain endothelial cell cultures resulted in both higher TEER and better maintenance of permeability restriction over time [16]. Furthermore, GDNF addition can rescue barrier disruption caused by toxins and restore expression levels of claudin-5 and Zo-1 (also known as TJP1) [64]. Both the mRNA expression and the secretion of Ang-1 to the media were higher in LN521 astroglia compared to L2020 astroglia. We speculate that this could be the mechanism behind the increased expression of adherence junction protein VE-cadherin in r-iBECs cocultured with astroglia differentiated on LN521 compared to L2020. In addition, mRNA levels of GDNF were higher in astroglia differentiated on LN521, which could be another contributing factor to higher expression of VE-cadherin in r-iBECs cocultured with astroglia differentiated on LN521. However, the GDNF concentration in the media was very low and differences between astroglia differentiated on LN521 and L2020 could therefore not be evaluated. No differences in mRNA levels of Zo-1 were detected. It is possible that higher Ang-1 levels affect Zo-1 stability in accordance with previous studies [63], however to prove such affects permeability challenge experiments would have to be performed. r-iBECs in coculture with L2020 astroglia had lower fluorescein permeability than r-iBECs in coculture with LN521 astroglia. In this study, markers for paracellular transport (TEER) and transcytosis (CAV1) across r-iBECs were investigated. CAV1 encodes for caveolin1, a protein needed for the formation of transcytotic vesicles. To understand differences in fluorescein permeability additional evaluation of permeability and barrier properties needs to be performed, for example, there could be differences in the transcytosis rate not detectable through studying only mRNA expression of CAV1.

Another interesting observation is that the increased expression of ABCB1 and the decreased expression of CAV1 in r-iBECs appear to be dependent on coculture with differentiated astroglia, as it did not appear in coculture with NES. ABCB1 encodes for P-gp and CAV1 encodes for caveolin1, increased P-gp and decreased caveolin1 both restrict the permeability across the brain endothelium. P-gp through increased efflux of small lipophilic molecules and decreased caveolin1 by reduced transcytosis, both high P-gp and low caveolin1 are hallmarks of brain endothelium. It is still unclear whether the maturation state of astroglia in coculture with endothelial cells influences the barrier properties. Published in vitro BBB coculture models use neural stem cells or fetal astroglial cells [37, 38] and, more recently, differentiated astrocytes [34, 39, 65]. Our results suggest that coculture with differentiated astroglia effects protein expression in r-iBECs important for brain endothelium specific permeability restriction.

## Conclusions

We evaluated astroglia generated on two different laminins: human-defined laminin LN521 and murine laminin L2020, using three different donors of hiPSC. In these experiments, we have shown that astroglia of good quality can be generated from hiPSC on both LN521 and L2020. This work provides a starting point for defined and xeno-free production of hiPSC-derived astroglia for BBB coculture models. Astroglia derived on LN521 can be used in hiPSC-derived BBB models in vitro with similar results to L2020 derived astroglia. In addition, our results suggest that astroglia differentiated on LN521 have a higher secretion of factors important for BBB formation in hiPSC-derived endothelial cells such as Ang-1.

## Additional file

**Additional file 1: S1.** Antibodies. **S2.** qPCR assays.

## Data Availability

All data generated or analysed during this study are included in this published article and its additional files.

## Abbreviations

ABCB1: ATP-binging casette transporter family B transporter 1
ABCG2: ATP-binging casette transporter family G transporter 2
AD: Alzheimer’s disease
ALDH1L1: aldehyde dehydrogenase 1 L1
ALS: amyotrophic lateral sclerosis
Ang-1: angiopoietin-1
APOE: apolipoprotein E
ASCT2: alanine, serine, cysteine transporter 2
BBB: blood–brain barrier
BM: basement membrane
BMEC: brain microvascular endothelial cells
CAV1: caveolin1
DAPI: 4′,6-diamidino-2-phenylindole
EAAT1: excitatory amino acid transporter 1
EAAT2: excitatory amino acid transporter 2
FABP7: fatty acid binding protein 7
FBS: fetal bovine serum
GABA: gamma-aminobutyric acid
GAPDH: glyceraldehyde 3-phosphate dehydrogenase
GDNF: glial derived neurotrophic factor
GFAP: glial fibrillary acid protein
GLUL: glutamine synthetase
GMP: good manufacturing practice
HBSS: Hank’s balanced salt solution
hiPSC: human induced pluripotent stem cells
IL6: interleukin 6
IL8: interleukin 8
L2020: laminin from murine Engelbreth-Holm-Swarm sarcoma
LN521: laminin-521
NES: long-term neuroepithelial stem cells
P-gp: P-glycoprotein
PBS: phosphate buffered saline
PD: Parkinson’s disease
r-iBECs: hiPSC-derived brain endothelial cells from r-iPSC1 J line
S100B: S100 calcium-binding protein B
SLC1A2: solute carrier family 1 member 2
SNAT3: sodium-coupled neutral amino acid transporter 3
SNAT5: sodium-coupled neutral amino acid transporter 5
TEER: trans endothelial electrical resistance
TJP1: tight junction protein 1
TJP3: tight junction protein 3
VE-cadherin: vascular endothelial cadherin
vWF: von Willebrand factor
Zo-1: zonula occludens 1
