# Supplementary material for: Enhanced xeno-free differentiation of hiPSC-derived astroglia applied in a blood–brain barrier model
Source: Fluids Barriers CNS. 2019 Aug 29;16:27. doi: 10.1186/s12987-019-0147-4 (PMC6714544; doi:10.1186/s12987-019-0147-4)
Supplement: Supplementary file 1 — Additional file 1: S1. Antibodies. S2. qPCR assays. [file 12987_2019_147_MOESM1_ESM.docx]

Additional file

| S1. Antibodies | |  | |  |  |  |
| --- | --- | --- | --- | --- | --- | --- |
| Primary antibody target | | Species | | Supplier | Cat. No. | Dilution |
| FABP7 | | Rabbit | | Merck Millipore | ABN14 | 1:250 |
| GFAP | | Rabbit | | Merck Millipore | AB5804 | 1:1500 |
| S100B | | Mouse | | Abcam | ab11179 | 1:500 |
| GLAST | | Mouse | | Miltenyi Biotec | 130-095-822 | 1:100 |
| Secondary antibody | | Species | | Supplier | Cat. No. | Dilution |
| Goat anti-rabbit IgG | | Goat | | Thermo Fisher | A-11012 | 1:1000 |
| Goat anti-mouse IgG | | Goat | | Thermo Fisher | A-11001 | 1:1000 |
|  | |  | |  |  |  |
| S2. qPCR assays | | |  |  |  |  |
| Target mRNA | TaqMan Assay ID | |  |  |  |  |
| GFAP | HS00909236_m1 | |  |  |  |  |
| S100B | HS00902901_m1 | |  |  |  |  |
| Nestin | HS04187831_g1 | |  |  |  |  |
| CD44 | Hs01075861_m1 | |  |  |  |  |
| FABP7 | Hs00361424_g1 | |  |  |  |  |
| Aldh1L1 | HS00201836_m1 | |  |  |  |  |
| GDNF | Hs01931883_s1 | |  |  |  |  |
| Ang1 | Hs00919202_m1 | |  |  |  |  |
| GLAST | hs00188193_m1 | |  |  |  |  |
| GLT1 | hs01102423_m1 | |  |  |  |  |
| SNAT3 | Hs01006103_m1 | |  |  |  |  |
| SNAT5 | Hs01012028_m1 | |  |  |  |  |
| ASCT2 | HS01056542_m1 | |  |  |  |  |
| GLUL | HS00365928_g1 | |  |  |  |  |
| SLC6A1 | Hs01104475_m1 | |  |  |  |  |
| SLC1A2 | HS00892681_m1 | |  |  |  |  |
| VE-Cadherin | HS00901465_m1 | |  |  |  |  |
| ABCA1 | HS00184500_m1 | |  |  |  |  |
| CD31 | HS01065282_m1 | |  |  |  |  |
| ABCG2 | HS01053790_m1 | |  |  |  |  |
| TJP1 | HS01551861_m1 | |  |  |  |  |
| vWF | HS01109446_m1 | |  |  |  |  |
| TJP3 | HS00274276_m1 | |  |  |  |  |
| Cav1 | HS00971716_m1 | |  |  |  |  |
| GAPDH | HS02758991_g1 | |  |  |  |  |
